# Prognostic Implications of Left Ventricular Global Longitudinal Strain in Patients With Surgically Treated Mitral Valve Disease and Preserved Ejection Fraction

**DOI:** 10.3389/fcvm.2021.775533

**Published:** 2022-01-20

**Authors:** Seon Hwa Lee, Purevjargal Lhagvasuren, Jiwon Seo, Iksung Cho, Dae-Young Kim, Geu-Ru Hong, Jong-Won Ha, Chi Young Shim

**Affiliations:** Division of Cardiology, Severance Cardiovascular Hospital, Yonsei University College of Medicine, Seoul, South Korea

**Keywords:** left ventricular global longitudinal strain/LV-GLS, outcome, preserved left ventricular ejection fraction, post-operation, mitral valve surgery

## Abstract

**Background:**

This study investigated whether left ventricular (LV) global longitudinal strain (LV-GLS), as an LV function parameter less affected by mitral valve (MV) repair or prosthesis, is associated with clinical outcomes in patients with surgically treated MV disease.

**Methods:**

Among 750 patients who underwent MV surgery, we assessed LV-GLS by speckle tracking echocardiography in 344 patients (148 men, mean age 58 ± 13 years) who showed preserved LV ejection fraction on echocardiography between 6 months and 2 years after MV surgery and who did not undergo aortic valve surgery. The assessed clinical events included admission for worsening of heart failure and cardiac death.

**Results:**

During a period of 42.4 ± 26.0 months, 32 (9.3%) patients were hospitalized for worsening heart failure, and 3 (0.8%) died due to cardiac causes. The absolute value of LV-GLS (|LV-GLS|) was significantly lower in patients with clinical events than in those without (12.1 ± 3.1 vs. 15.0 ± 3.2%, *p* < 0.001) despite comparable LV ejection fraction between groups. |LV-GLS| showed predictive value for clinical events (cut-off 13.9%, area under the curve 0.744, *p* < 0.001). Patients with |LV-GLS| ≤14.0% had poorer outcomes than those with |LV-GLS| >14.0% (log-rank *p* < 0.001). Prognosis was worse in patients with |LV-GLS| ≤14.0% and pulmonary hypertension than among those who with |LV-GLS| ≤14.0% without pulmonary hypertension (log rank *p* < 0.001). In nested Cox proportional hazard regression models, reduced |LV-GLS| was independently associated with the occurrence of clinical events.

**Conclusions:**

In patients with surgically treated MV and preserved LV ejection fraction, assessment of LV-GLS provides functional information associated with cardiovascular outcomes.

## Synopsis

- Reduced |LV-GLS| had a significant and independent association with cardiovascular events in patients with surgically treated mitral valve (MV) disease and preserved ejection fraction (EF).
- Reduced |LV-GLS| and pulmonary hypertension showed worse outcomes compared with preserved |LV-GLS| or reduced |LV-GLS| without pulmonary hypertension.
- Assessment of LV-GLS provides functional information associated with cardiovascular outcomes in patients with surgically treated MV disease and preserved EF.

## Introduction

Readmission due to heart failure (HF) and cardiovascular events is not uncommon in patients with surgically treated mitral valve (MV) disease, even in those with preserved ejection fraction (EF) (1, 2). However, conventional echocardiographic parameters used to assess the risk of HF are not generally applicable in this population. In particular, the diagnosis of left ventricular (LV) diastolic dysfunction after MV surgery is challenging because many Doppler parameters are affected by the surgically treated MV (3). That is, the E/e' ratio does not reliably reflect LV filling pressure, and left atrial (LA) volume index is also more affected by MV disease before and after surgical correction than by LV diastolic dysfunction (4). Moreover, these parameters are also influenced by other factors, such as prosthetic valve regurgitation or obstruction, which can lead to increased LA pressure after surgery.

LV global longitudinal strain (LV-GLS) assessed by speckle tracking echocardiography is a useful parameter for detecting subclinical LV dysfunction in patients with preserved EF. It provides prognostic information for various cardiovascular diseases (5). In patients with native MV regurgitation, pre-operative LV-GLS can predict LV reverse remodeling and cardiovascular events after MV surgery (6–9). However, there is a paucity of data on the prognostic implications of post-operative LV-GLS in patients with surgically treated MV. In the present study, we hypothesized that post-operative LV-GLS would be associated with clinical outcomes in patients with preserved EF after MV surgery. To test our hypothesis, we assessed LV-GLS using speckle tracking echocardiography in patients with preserved EF on echocardiography between 6 months and 2 years after MV surgery.

## Materials and Methods

### Study Population

This study included a total of 750 patients who underwent MV surgery for the treatment of symptomatic severe MV disease from January 2010 to September 2019 at a single tertiary hospital. In patients with cardiac valve surgery, echocardiography is performed as part of the clinical routine at baseline before surgery and every 6 months or 1 year after surgery at the clinicians' discretion. We excluded patients who had undergone prior or concomitant aortic valve surgery (*n* = 200), those who did not undergo echocardiography between 6 months and 2 years after MV surgery (*n* = 137), those who presented with a LV ejection fraction (LVEF) <50% (*n* = 34) or residual moderate or severe mitral regurgitation (MR) and MV obstruction (*n* = 2) on echocardiography performed between 6 months and 2 years after MV surgery, and those with a follow-up period of <6 months after echocardiography (*n* = 25). We also excluded cases involving poor image quality for strain measurement (*n* = 6). Finally, the analyses included 344 patients (Supplementary Figure 1). All echocardiography in this study was performed during the regular follow-up period, not during hospitalization for HF aggravation.

Patient clinical data, including data on medication history, demographics, and laboratory parameters, were recorded at the time of echocardiography performed between 6 months and 2 years after MV surgery and obtained from hospital records. We also evaluated possible prognostic factors as Charlson comorbidity index (CCI) (10). CCI was calculated as the sum of scores for several comorbidities based on the original definition. When defining AF, rhythm estimation was performed if AF was documented on electrocardiogram regardless of the rhythm at the time of echocardiography. Subgroup analyses were performed based on the rhythm characteristics [sinus rhythm (*n* = 139) vs. atrial fibrillation (*n* = 205)]. We also performed subgroup analyses according to follow-up duration after echocardiography [follow-up duration <40 months (*n* =172) vs. follow-up duration ≥40 months (*n* = 172)]. The study protocol was developed according to the principles of the Declaration of Helsinki, and was approved by the Institutional Review Board of Severance Hospital. Informed consent was waived for the retrospectively analyzed patients.

### Echocardiography

All patients underwent two-dimensional Doppler echocardiography and speckle tracking echocardiography using a standard ultrasound machine (Vivid E9; GE Medical Systems; Wauwatosa, WI, Philips iE33; Philips Healthcare; Netherlands) with a 2.5–3.5 MHz probe. Standard echocardiographic measurements were performed according to recommendations from the American Society of Echocardiography guidelines (11). LVEF was measured using the biplane method of disks (modified Simpson's rule) from apical four- and two-chamber views (11). LA volume index was assessed manually using Simpson's method at the end of the ventricular systole and indexed to the body surface area (11). Systolic (S') and peak early (e') and late (A') diastolic annular velocities were obtained via tissue Doppler imaging of the septal mitral annulus. Pulmonary artery systolic pressure (PASP) was estimated using the following formula: 4 × [tricuspid regurgitant velocity (m/s)]^2^ + right atrial pressure (mmHg). Right atrial pressure was estimated from the inferior vena cava (IVC) diameter and respiratory variation as follows: 5 mmHg (IVC ≤2.1 cm, collapse with sniff >50%), 10 mmHg (IVC>2.1 cm, collapse >50%), and 15 mmHg (IVC >2.1 cm, collapse with sniff <50%) (12). Comprehensive assessment of prosthetic MV and repaired MV function was evaluated based on imaging and Doppler parameters: measurement of effective orifice area, Doppler velocity index, peak transvalvular velocity-time integral, and pressure half-time (13).

### Speckle Tracking Echocardiography

**Figure 1:**
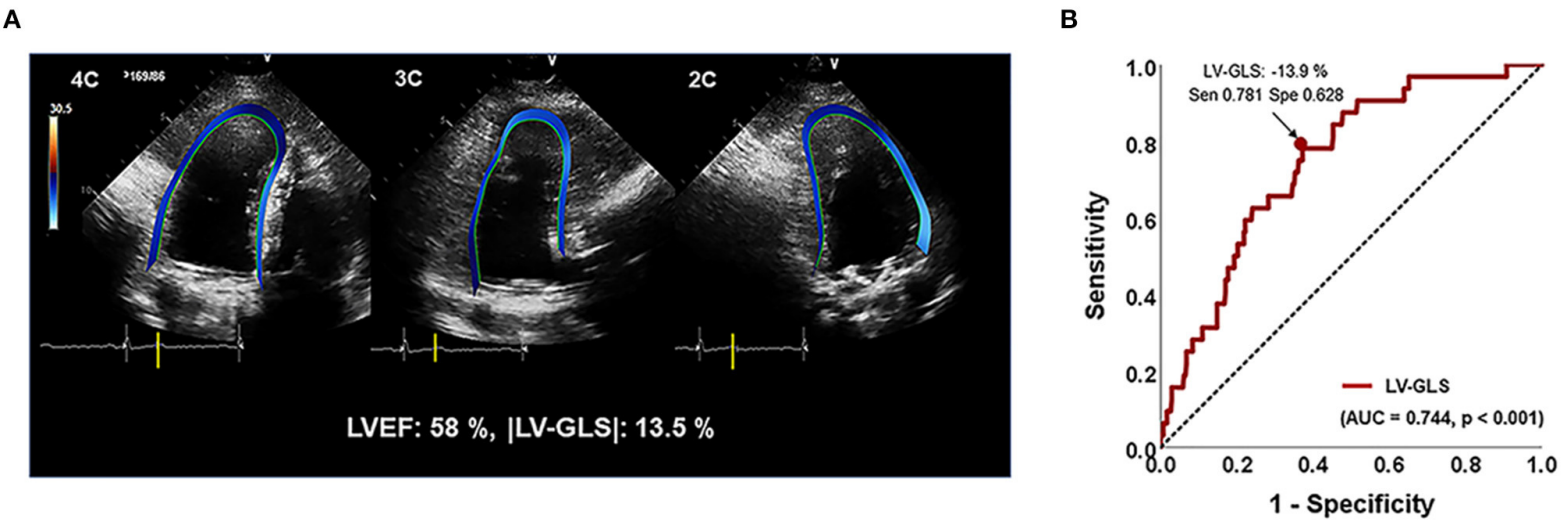
**(A)** Representative images for assessing LV-GLS. **(B)** Area under the receiver-operating characteristics curve (area under the curve) of |LV-GLS|. |LV-GLS|, the absolute value of left ventricular longitudinal strain. The Arrow indicated that the position of the value of sensitivity and specificity of 78% and 63% when |LV-GLS| has largest AUC.

From two-dimensional images of the apical two-, three-, and four-chamber views, LV-GLS was measured offline using a vendor-independent software package (TomTec software; Image Arena 4.6, Munich, Germany), as described previously (14). For myocardial deformation analysis, the endocardial border was traced on the end-systolic frame in each selected image. The end-systolic frame was defined by the QRS complex or based on the smallest LV volume during the cardiac cycle. The software automatically tracked speckles along the endocardial border and myocardium throughout the cardiac cycle. The value for LV-GLS was obtained by averaging all segmental strain values from three apical views (Figure 1A). LV-GLS was analyzed for a single cardiac cycle in patients with sinus rhythm. In patients with AF, we selected an index beat method for the measurement of LV-GLS, which has been validated for LV-GLS in a previous study (15). |LV-GLS| was defined as the absolute value of post-operative LV-GLS (removing the conventional negative value of GLS data). |LV-GLS|_preop_ was used to represent absolute value of pre-operative LV-GLS. Echocardiographic data and strain values were analyzed by an experienced cardiologist blinded to clinical data. We randomly selected 20 patients from the study population and analyzed the intra- and inter-observer reproducibility of LV-GLS measurement by Bland–Altman analysis. The intra-class correlation coefficients for |LV-GLS| were 0.978 and 0.962 with regard to intra- and inter-observer variation, respectively. The Bland–Altman analysis showed the limits of agreement (LOA) across a broad range of GLS values; the bias for intra- and inter-observer measurements of LV-GLS were 0.46% (range: −1.02% to 1.03%, 95% LOA) and 0.45% (range: −1.25% to 1.05%), respectively.

### Clinical Events

The clinical events in this study were death from cardiovascular causes and hospitalization for HF after echocardiography between 6 months and 2 years after MV surgery. Our study population was followed up until August 2020. Death from cardiovascular causes was defined as death due to HF, arrhythmia, acute coronary syndrome, stroke, and sudden cardiac death. Hospitalization for HF was defined as readmission due to worsening of symptoms or signs of HF.

### Statistical Analysis

Continuous variables are presented as mean ± standard deviation (SD) or median (interquartile range) and were compared using the paired Student's *t*-test (for normally distributed data) or the Mann–Whitney *U* test (for non-normally distributed data). Categorical variables are presented as absolute numbers and percentages and were analyzed using the chi-square or Fisher's exact test. The cut-off values for CCI and |LV-GLS| were determined as the values that maximized the sum of the sensitivity and specificity for clinical events in receiver operating characteristic (ROC) curve analysis. The cumulative event-free survival was calculated using Kaplan–Meier curves and compared between groups by log-rank tests. Cox univariate analysis was performed to determine the relationships between clinical and echocardiographic variables and clinical events. Nested Cox proportional hazard regression models were used to estimate the risk of cardiovascular events according to |LV-GLS|. Initially, |LV-GLS| was included in Cox proportional hazards regression as a covariate, with adjustments for CCI and AF. In further multivariate analysis, we adjusted for LAVI, PASP, and pre-operative LV-GLS. The variables selected for entry into multivariate analysis were those with a *p*-value < 0.1 in Cox univariate analysis. A two-sided *p*-value < 0.05 was considered to indicate statistical significance. All analyses were performed using IBM SPSS Statistics for Windows, version 25.0 (IBM Corp., Armonk NY, USA).

## Results

### Baseline Characteristics and Clinical Events

The baseline characteristics of the study population according to the occurrence of clinical events are presented in Table 1. The mean age was 58 ± 13 years, and 57% of patients were female. Patients who experienced clinical events had more comorbidities, such as atrial fibrillation, diabetes mellitus, and chronic kidney disease, and had been taking more cardiovascular drugs. There were 214 (62.2%) and 130 (37.8%) patients with severe MR and severe MS, respectively. In the patients with severe MR, all patients had primary MR without secondary MR. Among these patients, 173 (81.2%) patients had prolapse or flail of MV and 31 (14.4%) patients had rheumatic MV and 10 (4.6%) patients had barlow MV. In total, 224 (64.2%) patients in our study population underwent MV replacement and 125 (35.8%) underwent MV repair. There were no significant differences between the two groups in surgery type, percentage of concomitant surgery, or laboratory parameters. During 42.4 ± 26.0 months of follow-up after LV-GLS assessment, 32 (9.3%) patients were hospitalized for worsening HF, and 3 (0.8%) died due to cardiac causes.

**Table 1:** Baseline characteristics of the study population.

**Variable**	**Total (***n*** = 344)**	**Without clinical events (***n*** = 312)**	**With clinical events (***n*** = 32)**	***p*** **-value**
Age, years	58 ± 13	58 ± 13	61 ± 10	0.091
Female sex, *n* (%)	196 (57)	175 (56.1)	21 (65.6)	0.299
Body mass index, kg/m^2^	23.6 ± 4.3	23.3 ± 3.2	23.7 ± 5.6	0.682
Hypertension, *n* (%)	184 (53.5)	165 (52.9)	19 (59.4)	0.483
Diabetes mellitus, *n* (%)	59 (17.2)	46 (14.7)	13 (40.6)	<0.001
Dyslipidemia, *n* (%)	169 (49.1)	148 (47.4)	21 (65.6)	0.050
CAD, *n* (%)	28 (8.1)	25 (8.0)	3 (9.4)	0.574
CKD, *n* (%)	25 (7.3)	15 (4.8)	10 (31.3)	<0.001
Atrial fibrillation, *n* (%)	205 (59.6)	178 (57.1)	27 (84.4)	0.003
Serum creatinine, mg/dl	0.82 ± 0.36	0.79 ± 0.21	1.08 ± 0.90	0.081
Hemoglobin, g/dL	12.6 ± 2.2	12.6 ± 2.2	12.3 ± 2.4	0.395
**Underlying MV function,** ***n*** **(%)**				
Severe MR	214 (62.2)	200 (64.1)	14 (43.8)	0.024
Severe MS	130 (37.8)	112 (35.9)	18 (56.3)	
**Type of MV surgery**, ***n*** **(%)**				
MV replacement	224 (64.2)	196 (62.8)	25 (78.1)	0.085
MV repair	125 (35.8)	116 (37.2)	7 (21.9)	
**Concomitant surgery**, ***n*** **(%)**				
TAP	152 (44.2)	135 (43.0)	17 (53.1)	0.486
CABG	13 (3.8)	10 (3.2)	3 (9.4)	0.082
Maze	94 (27.3)	84 (26.9)	10 (31.3)	0.601
**Medications**, ***n*** **(%)**				
ACE-I or ARB	93 (27)	87 (27.9)	6 (18.8)	0.268
Beta-blocker	88 (25.6)	81 (26.0)	7 (21.9)	0.614
Aldosterone antagonist	57 (16.6)	44 (14.1)	13 (40.6)	<0.001
Loop diuretic	137 (39.8)	118 (37.8)	19 (61.3)	0.011

*CAD, coronary artery disease; CKD, chronic kidney disease; MV, mitral valve; MR, mitral regurgitation; MS, mitral stenosis; TAP, tricuspid annuloplasty; CABG, coronary artery bypass graft; ACEI, angiotensin converting enzyme inhibitor; ARB, angiotensin receptor blocker*.

### Echocardiographic Characteristics and |LV-GLS|

Table 2 shows conventional echocardiographic parameters, pre-operative LV-GLS, and post-operative LV-GLS assessed between 6 months and 2 years after MV surgery. The mean pre-operative |LV-GLS| was 16.1 ± 4.8%, which was higher than |LV-GLS| after MV surgery. Patients who experienced clinical events had a significantly lower |LV-GLS|_preop_ than those who did not (14.1 ± 3.8% vs. 16.3 ± 4.8%, *p* = 0.018). There were no differences in the timing of post-operative echocardiography and clinical follow-up duration after echocardiography between patients with and without clinical events.

**Table 2:** Echocardiographic parameters and left ventricular global longitudinal strain.

	**Total (***n*** = 344)**	**Without clinical events (***n*** = 312)**	**With clinical events (***n*** = 32)**	***p*** **-value**
LVEF_preop_. %	64.4 ± 8.4	64.7 ± 8.4	61.7 ± 8.3	0.064
|LV-GLS|_preop_. %	16.1 ± 4.8	16.3 ± 4.8	14.1 ± 3.8	0.018
Timing of post-operative echo, months	12.0 ± 4.9	11.9 ± 4.0	13.2 ± 4.3	0.177
Follow-up after echo, months	42.4 ± 26.0	42.2 ± 25.3	44.9 ± 32.7	0.575
LVEDD, mm	48.4 ± 5.2	48.4 ± 5.0	47.6 ± 10.5	0.774
LVESD, mm	32.8 ± 4.6	32.7 ± 4.4	33.5 ± 5.9	0.455
LVEF, %	63.3 ± 6.5	63.3 ± 6.6	62.6 ± 5.9	0.550
LAVI, mL/m^2^	59.7 ± 36.2	57.9 ± 35.8	77.6 ± 35.8	0.003
LVMI, g/m^2^	96.7 ± 25.7	95.6 ± 23.5	110.7 ± 40.1	0.048
S', cm/sec	6.0 ± 1.4	6.0 ± 1.4	5.5 ± 1.4	0.101
e' cm/sec	6.1 ± 1.8	6.1 ± 1.8	5.2 ± 2.1	0.015
A', cm/sec	6.0 ± 2.1	6.0 ± 2.1	4.9 ± 2.0	0.113
PASP, mmHg	29.6 ± 11.7	28.2 ± 8.5	44.4 ± 23.9	0.001
MV MDPG, mmHg	3.7 ± 2.1	3.6 ± 2.1	4.1 ± 2.1	0.194
|LV-GLS|, %	14.8 ± 3.3	15.0 ± 3.2	12.1 ± 3.1	<0.001

*LVEF_preop_, pre-operative left ventricular ejection fraction; |LV-GLS|_preop_, pre-operative absolute value of left ventricular global longitudinal strain; LVEDD, left ventricular end-diastolic dimension; LVESD, left ventricular end-systolic dimension; LVEF, left ventricular ejection fraction; LAVI, left atrial volume index; S', systolic mitral annular tissue Doppler velocity; e', early diastolic mitral annular tissue Doppler velocity; A', late diastolic mitral annular tissue Doppler velocity; PASP, pulmonary artery systolic pressure; MV, mitral valve; MDPG, mean diastolic pressure gradient; |LV-GLS|, absolute value of left ventricular global longitudinal strain*.

**Figure 2:**
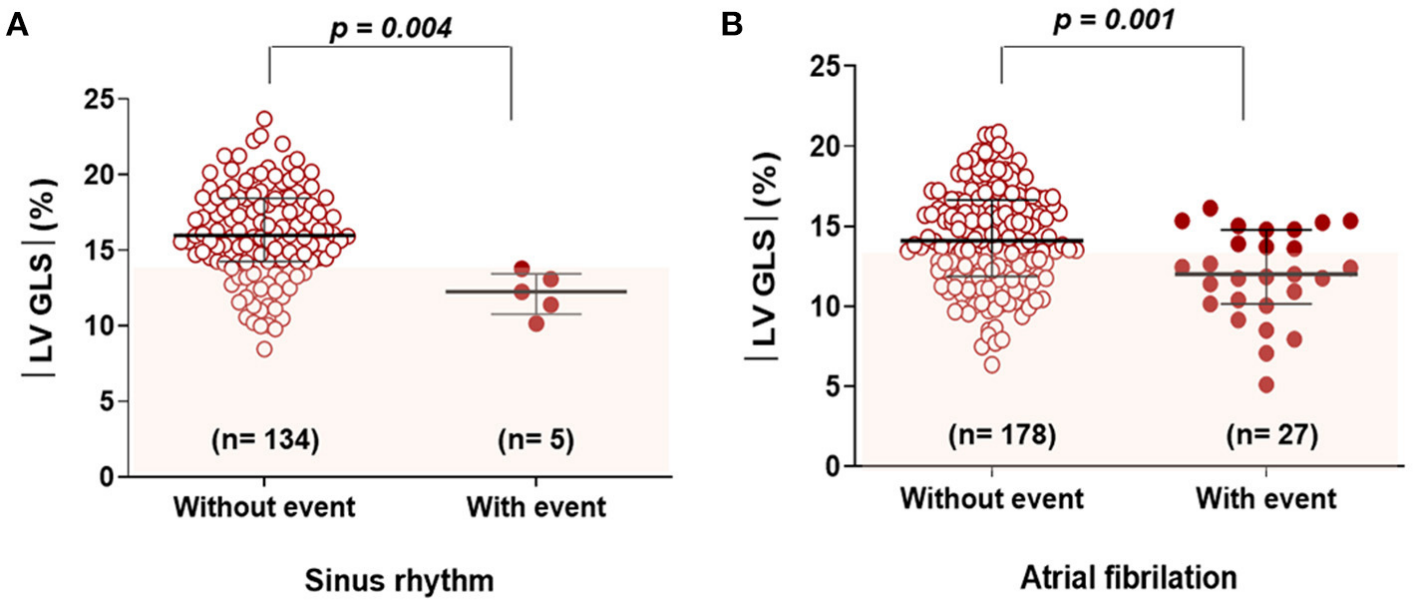
Comparison of |LV-GLS| according to clinical events in subgroups categorized by rhythm **(A)** and **(B)**. |LV-GLS|, the absolute value of left ventricular longitudinal strain.

Since this study excluded patients with reduced LVEF, the average LVEF was 63.3%. There were no significant differences in LVEF between patients with and without clinical events; however, patients who experienced clinical events had a significantly higher LA volume index, LV mass index, and PASP. In terms of tissue Doppler parameters, e' velocity was lower in patients with clinical events than in patients without clinical events. The mean |LV-GLS| was 14.8 ± 3.3%. Patients who experienced clinical events had a significantly lower |LV-GLS| compared to patients who did not (12.1 ± 3.1% vs. 15.0 ± 3.2% *p* < 0.001). Regardless of sinus rhythm or atrial fibrillation, |LV-GLS| was significantly lower in patients with clinical events than in those without (Figures 2A,B).

### Prognostic Implications of LV-GLS

**Figure 3:**
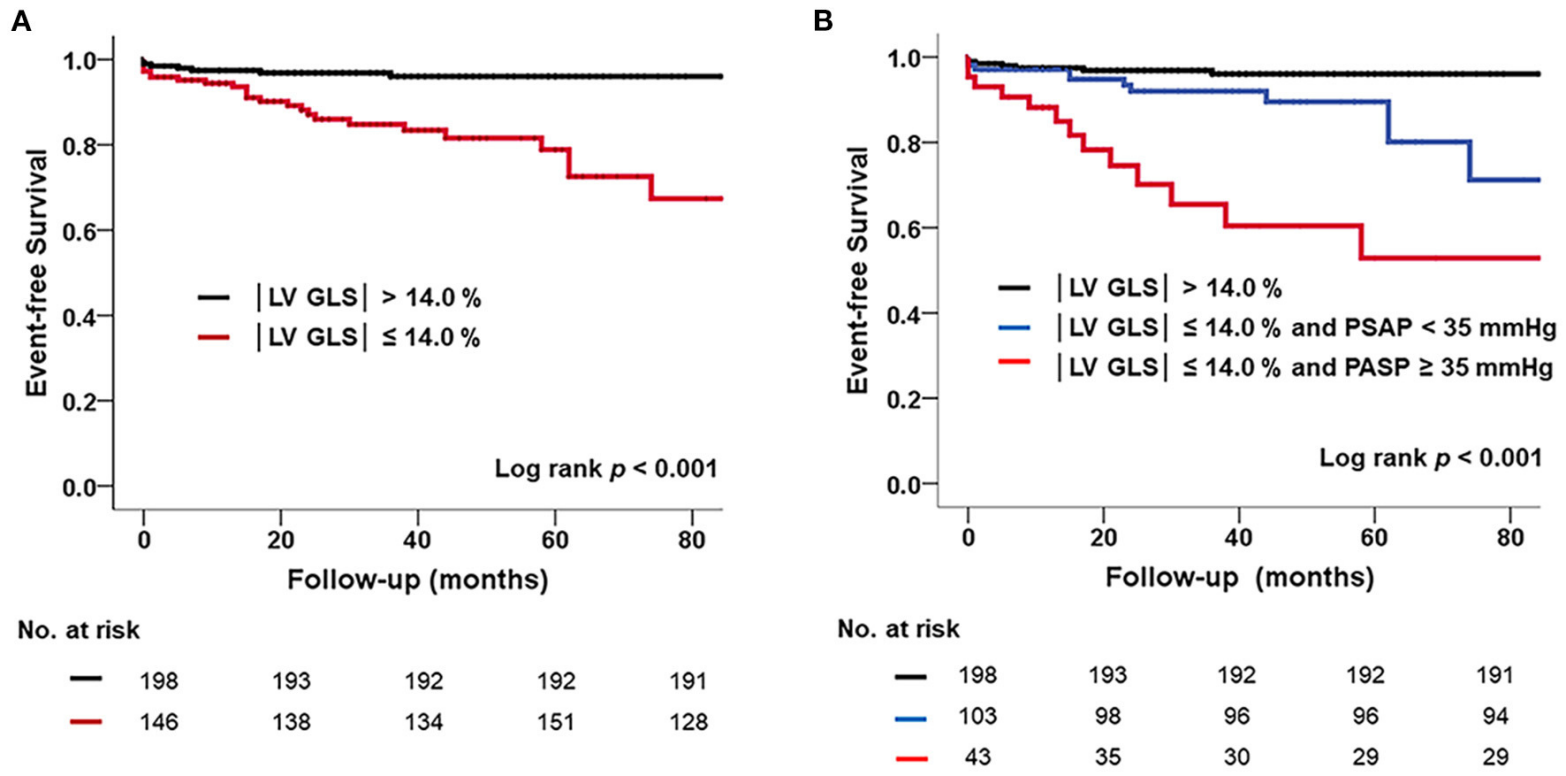
**(A)** Kaplan–Meier survival curve showing poor prognosis in subjects with reduced |LV-GLS| compared those without. **(B)** Poorer outcomes in subjects with reduced |LV-GLS| and pulmonary hypertension. |LV-GLS|, the absolute value of left ventricular longitudinal strain.

In ROC analysis, the |LV-GLS| value showing the largest area under the curve (AUC) for the association with adverse clinical outcomes was 13.9% (AUC 0.744, *p* < 0.001), with a sensitivity and specificity of 78 and 63%, respectively (Figure 1B). In the subgroups according to cardiac rhythm, when GLS 14% is set as a cut off value, the sensitivity and specificity of 74 and 51%, respectively in the patients with AF and the sensitivity and specificity of 80 and 78%, respectively in the patients with sinus rhythm. |LV-GLS| showed a largest AUC of predictive value for clinical events than conventional parameters (LAVI > 40 ml/m^2^, AUC = 0.604, *p* = 0.065; TR >2.8 m/s, AUC = 0.670, *p* = 0.003). After dividing patients into two groups based on the cut-off value, those with |LV-GLS| ≤14.0% had poorer outcomes than patients with |LV-GLS| >14.0% (log-rank *p* < 0.001; Figure 3A). When the subjects were classified into three groups according to |LV-GLS| and PASP, those with |LV-GLS| ≤14.0% and PASP ≥35 mmHg had significantly worse outcomes compared to those in the other groups (log-rank *p* < 0.001; Figure 3B). There was no significant correlation between |LV-GLS| value and post-operative echocardiography timing (*r* = 0.018, *p* = 0.742). In addition, if |LV-GLS| was lower than 14.0%, there were more clinical events irrespective of post-operative echocardiography timing (Supplementary Figure 2). Also, in subgroup analysis according to follow-up duration, patients with |LV-GLS| ≤14.0% consistently showed a worse prognosis (Supplementary Figure 3).

### Factors Associated With Clinical Events

Table 3 shows the results of univariate Cox regression analyses to investigate the factors associated with clinical events. In univariate survival analysis, diabetes mellitus, chronic kidney disease, atrial fibrillation, echocardiographic parameters such as e' velocity, LA volume index, PASP, |LV-GLS|_preop_, and |LV-GLS| were significantly associated with the occurrence of cardiac events. Lower |LV-GLS| (|LV-GLS| ≤ 14.0% and |LV-GLS| ≤ 16.0%) was independently associated with cardiac events after adjustment for conventional echocardiographic parameters (Table 4). In nested Cox proportional hazard regression models (Table 5), patients with lower |LV-GLS| had increased risk of adverse clinical outcomes in CCI and AF (Model1) adjusted analysis (hazard ratio, HR: 4.3; 95% confidence interval, CI: 1.83–10.29, *p* = 0.001). In further multivariate models, the associations were attenuated, and lower |LV-GLS| remained a significant factor in adverse clinical outcomes (Model 2; HR: 4.33; CI: 1.83–10.26; *p* = 0.001, Model 3; HR: 3.30; CI: 1.34–8.12; *p* =0.009). Moreover, lower |LV-GLS| was significantly associated with adverse clinical outcomes, even after adjusting for |LV-GLS|_preop_ (Model 4; HR: 3.68; CI: 1.54–8.80; *p* = 0.003). Also, there were no differences in pre and post-operative change in LVGLS between patients without clinical events and those with clinical events (1.2 ± 4.9 vs. 1.8 ± 4.7, *p* = 0.500).

**Table 3:** Cox proportional hazard regression univariate analysis of clinical outcomes.

	**HR (95% CI)**	***P***
Age	1.027 (0.997–1.058)	0.081
Female sex	1.396 (0.667–2.920)	0.376
Hypertension	1.253 (0.618–2.543)	0.531
Diabetes mellitus	3.076 (1.510–6.266)	0.002
CKD	7.995 (3.747–17.06)	<0.001
CAD	1.513 (0.388–2.294)	0.898
Charlson comorbidity index>1	5.889 (1.185–7.046)	0.001
Atrial fibrillation	3.874 (1.487–10.094)	0.006
S'	0.775 (0.582–1.031)	0.080
e'	0.724 (0.567–0.924)	0.010
LAVI	1.009 (1.003–1.015)	0.005
LAVI > 34 ml/m^2^	4.230 (1.007–17.763)	0.049
LAVI > 40 ml/m^2^	3.750 (1.310–10.736)	0.014
LVEF	0.988 (0.939–1.039)	0.638
PASP	1.063 (1.045–1.081)	<0.001
TR > 2.8 m/s	5.236 (2.507–11.046)	<0.001
TR > 3.0 m/s	7.144 (3.357–15.205)	<0.001
|LV-GLS|_preop_	0.912 (0.842–0.988)	0.024
|LV-GLS|	0.772 (0.694–0.859)	<0.001
|LV-GLS| ≤ 14.0%	5.787 (2.490–13.450)	<0.001
|LV-GLS| ≤ 16.0%	5.601 (1.702–18.429)	0.005

*HR, hazard ratio; CI, confidence interval; CKD, chronic kidney disease; CAD, coronary artery disease; S', systolic mitral annular tissue Doppler velocity; e', early diastolic mitral annular tissue Doppler velocity; LAVI, left atrial volume index; LVEF, left ventricular ejection fraction; PASP, pulmonary artery systolic pressure; TR, tricuspid regurgitation; |LV-GLS|, absolute value of left ventricular global longitudinal strain*.

**Table 4:** Prognostic values of LV-GLS with conventional echocardiographic parameters for adverse outcomes by cox proportional hazard regression multivariate analysis according to different cut off value.

	**HR (95% CI)**	***P***
**Criteria 1**		
|LV-GLS| ≤ 14.0%	3.857 (1.613–9.223)	0.001
TR > 2.8 m/s	3.941 (1.861–8.347)	<0.001
LAVI > 34 ml/m^2^	2.285 (0.531–9.836)	0.267
**Criteria 2**		
|LV-GLS| ≤ 16.0%	3.679 (1.90–12.423)	0.009
TR > 3.0 m/s	4.109 (2.322–11.241)	0.001
LAVI > 40 ml/m^2^	1.689 (0.553–5.157)	0.357

**Table 5:** Cox proportional hazards model of clinical outcomes (nested analysis).

	**Model 1**		**Model 2**		**Model 3**		**Model 4**	
	**HR (95% CI)**	***P***	**HR (95% CI)**	***P***	**HR (95% CI)**	***P***	**HR (95% CI)**	***P***
|LV-GLS| > 14.0%	Reference		Reference		Reference		Reference
|LV-GLS| ≤ 14.0%	4.336 (1.827–10.289)	0.001	4.330 (1.828–10.258)	0.001	3.302 (1.343–8.122)	0.009	3.681 (1.540–8.801)	0.003

*Model 1: Adjusted Charlson comorbidity index>1 and AF*.

*Model 2: Adjusted Charlson comorbidity index>1, AF, and LAVI*.

*Model 3: Adjusted Charlson comorbidity index>1, AF, LAVI, and PASP*.

*Model 4: Adjusted Charlson comorbidity index>1, AF, LAVI, and |LV-GLS|_preop_*.

### Incremental Prognostic Value of LV-GLS

**Figure 4:**
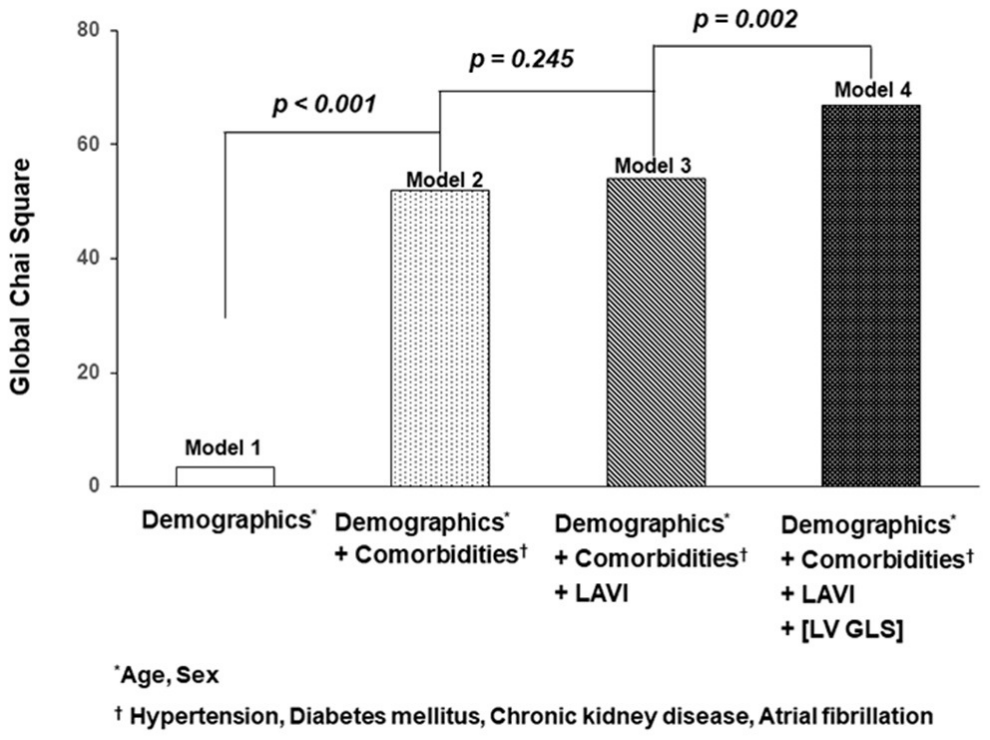
Incremental value of |LV-GLS|. The graphs show the incremental value of |LV-GLS| according to demographics, clinical factors, and LAVI. |LV-GLS|, the absolute value of left ventricular longitudinal strain; LAVI, left atrial volume index.

To evaluate incremental prognostic value of LV-GLS on cardiovascular events, we used global chi-square testing (Figure 4). Model 1 was adjusted for age and sex. In model 2, in which comorbidities including hypertension, diabetes mellitus, chronic kidney disease and atrial fibrillation were added to Model 1, the value increased to significantly (*p* < 0.001). Model 3 adjustments included the factors in Model 2 and LAVI with significantly elevated prognostic value. Finally, in Model 4, |LV-GLS| provided an incremental prognostic value with regard to the association with cardiac events (global chi-square from 54 to 69; *p* = 0.002).

## Discussion

The principal findings of this study were as follows: (1) reduced post-operative |LV-GLS| had a significant and independent association with cardiovascular events in patients with surgically treated MV disease and preserved EF; (2) patients with both reduced |LV-GLS| and pulmonary hypertension showed worse outcomes compared to patients with preserved |LV-GLS| or those with reduced |LV-GLS| without pulmonary hypertension. The results of this study suggest that |LV-GLS| is associated with adverse clinical outcomes. Furthermore, the presence of pulmonary hypertension is related to unfavorable outcomes in patients with surgically treated MV disease and preserved EF.

MV disease is one of the most common heart valve diseases, particularly in the aging population (16); moreover, HF with preserved EF is also prevalent and accounts for more than half of all HF hospitalizations (17, 18). Therefore, many patients may have both MV disease and HF, and risk stratification for HF with preserved EF is necessary even after the treatment for MV disease. In particular, with recent advances in surgical and percutaneous interventions for MV disease, interest in cardiac function and prognosis after the correction of MV disease is increasing. However, in patients with surgically treated MV disease, there are limitations to applying conventional echocardiographic parameters obtained via mitral inflow pulse wave Doppler and mitral annular tissue Doppler imaging with regard to risk stratification for HF. Owing to the MV prosthesis, the Doppler velocities from mitral inflow measurements are elevated compared to actual blood flow velocities (19, 20), and annular fixation of the prosthesis tends to decrease tissue Doppler velocities at the mitral annulus (21). Thus, the E/e' does not accurately reflect LV filling pressure. Moreover, the LA volume index is also more affected by the chronicity of underlying MV disease and atrial rhythm than by LV diastolic dysfunction. Due to these limitations, risk assessment depends only on LVEF, and no existing functional parameters can accurately predict the occurrence of HF and the prognosis of patients who have undergone MV surgery. In this study, patients with clinical events were more commonly prescribed aldosterone antagonists or loop diuretics. This fact indirectly indicates that these patients showed more signs and symptoms of HF despite having preserved LVEF and no problems with MV disease post-operative. Patients with treated MV disease may have subclinical or overt LV longitudinal dysfunction associated with various cardiovascular risk factors. Patients with surgically treated MV disease are not free from the occurrence of HF with preserved EF due to aging and risk factors. Since there is a history of MV disease, LV longitudinal dysfunction confirmed through LV-GLS can be a residual dysfunction of previous LV dysfunction. Whatever the mechanism is, evaluating LV longitudinal function in patients with previously treated MV disease is clinically important, and LV-GLS can be a diagnostic measure of HF with preserved EF and a useful indicator associated with clinical events. Furthermore, the stronger association of LV-GLS plus PASP with cardiovascular risk may contribute to better decision-making for further treatment in this population (4).

The importance of LV function as well as valve function itself has been emphasized in patients with MV disease, although the evaluation of LV function remains highly challenging in this population (22). LV-GLS is a more sensitive parameter of ventricular dysfunction in various diseases, especially in those involving preserved LVEF (23–25). Previous studies have suggested that early detection of subclinical LV dysfunction by speckle tracking echocardiography in asymptomatic patients with chronic MV disease is important to optimize the timing of MV interventions and to prevent LV dysfunction after surgery (26, 27). In recent studies, the cut-off value of LV-GLS before MV surgery was ~-18%, which could be used to predict clinical outcomes in patients with primary severe MR undergoing MV surgery (26, 27). In addition, the authors have also studied the time course of LV function by analyzing serial changes in LV-GLS before and after MV surgery (28). Until now, most studies have focused on the detection of LV dysfunction in the native valve state before MV surgery and the prediction of LV dysfunction after surgery (26, 27). However, the present study focused on the stratification of future cardiovascular risk by assessing LV-GLS in patients in whom MV disease had already been treated. The results of this study suggested that even if LVEF is preserved after MV surgery, the patient is at high risk for future HF-related hospitalization or cardiac death if |LV-GLS| is <14%. Moreover, in ROC analysis, |LV-GLS|_preop_ value showing the largest AUC for the association with adverse clinical outcomes was 15.0 % (ACU = 0.639, *p* = 0.011) with a sensitivity and specificity of 75 and 66%, respectively. Compared with the AUC of |LV-GLS| (AUC = 0.744, *p* < 0.001), there was no statistical difference (*p* = 0.121), but |LV-GLS| is more useful as a predictor of prognosis than |LV-GLS| _preop_ because of the large value of AUC in post-operative LVGLS. Also, reduced |LV-GLS| is significantly associated with cardiac events after adjusting for |LV-GLS|_preop_. The cut-off value of |LV-GLS| in our study was slightly lower than those in previous studies that assessed pre-operative status. This discrepancy might be mainly explained by the disappearance of volume overload and hemodynamic stabilization after MV surgery. In addition, the cut-off value of |LV-GLS| might be low because this study evaluated LV-GLS after successful MV surgery and after a certain recovery time; moreover, the study endpoint was not LV dysfunction, but rather clinical events during HF-related hospitalization or cardiac death.

In subgroup analysis according to rhythm characteristics, regardless of sinus rhythm or atrial fibrillation, |LV-GLS| remained significantly lower in patients with clinical events than in patients without clinical events. Atrial fibrillation is common in patients with MV disease; hence, this study included many patients with atrial fibrillation (59.6%). Patients with atrial fibrillation are at a high risk of HF with preserved EF.

In this study, estimated PASP was another important factor associated with clinical events. Patients with impaired |LV-GLS| associated with an increase in PASP had significantly worse outcomes than patients without an increase in PASP. The prognostic significance of pulmonary hypertension in left heart disease has been recognized in previous studies. The presence of pulmonary hypertension in left heart disease is associated with worse outcomes (29, 30). Furthermore, pulmonary hypertension with HF with preserved EF is common and is associated with mortality and cardiac hospitalization (28). In this respect, PASP may be a surrogate marker for cardiovascular events in patients with surgically treated MV disease with preserved EF.

This study presents clinical perspectives on treatment and future research. |LV-GLS| ≤14.0% and elevated PASP may be useful for diagnosing HF with preserved EF in patients with surgically treated MV disease.

This study has several limitations. First, the study population was evaluated retrospectively; thus, strain measurements were performed using stored images using vendor-independent software. For accurate measurement of strain, we excluded six patients with poor echocardiogram quality. Also, due to the limitations of retrospective studies, it possible that there were undetected confounding factors in multivariate analysis. Second, since the study population had been treated for MV disease, the number of patients with clinical events was small, which may be a limitation of this study. Third, PASP was calculated non-invasively by Doppler echocardiography and not measured by catheterization. Fourth, the difference by variable time point at which the post-operative echocardiography is present. Fifth, LA strain representing LA mechanical dysfunction is important prognostic value in patients undergoing MV surgery ADDIN EN.CITE (29–31). However, we did not evaluate LA mechanical function by speckle tracking echocardiography since there were technical limitations related to acoustic shadowing of the corrected MV. Therefore, we did not evaluate LA mechanical function in present study. Last, given that the cut-off value of LV-GLS might be different in other populations of surgically corrected MV, prospective validation in large populations is warranted.

## Conclusions

LV-GLS was significantly associated with cardiovascular events in patients with surgically treated MV disease with preserved LVEF. Our results suggest the potential prognostic implications of LV-GLS for risk stratification in patients with surgically treated MV disease with preserved EF.

## Data Availability Statement

The original contributions presented in the study are included in the article/Supplementary Material, further inquiries can be directed to the corresponding author/s.

## Author Contributions

SL and CS: planning, conducting the study, and drafting the manuscript. SL, PL, JS, IC, D-YK, G-RH, J-WH, and CS: collecting and interpreting data. CS: guarantor of the article. All authors contributed to the article and approved the submitted version.

## Funding

This study was supported in part by a faculty research grant from Yonsei University College of Medicine (6-2021-0096).

## Conflict of Interest

The authors declare that the research was conducted in the absence of any commercial or financial relationships that could be construed as a potential conflict of interest.

## Publisher's Note

All claims expressed in this article are solely those of the authors and do not necessarily represent those of their affiliated organizations, or those of the publisher, the editors and the reviewers. Any product that may be evaluated in this article, or claim that may be made by its manufacturer, is not guaranteed or endorsed by the publisher.

## Abbreviations

HF: Heart failure
MV: Mitral valve
EF: Ejection fraction
LV: Left ventricular
LA: Left atrial
LV-GLS: Left ventricular global longitudinal strain
LAVI: Left atrial volume index
AF: Atrial fibrillation
MR: Mitral regurgitation
MS: Mitral stenosis
S': Systolic mitral annular tissue Doppler velocity
e': Early diastolic mitral annular tissue Doppler velocity
A': Late diastolic mitral annular tissue Doppler velocity
PASP: Pulmonary artery systolic pressure.
