# Supplementary figures and images for: Prognostic Implications of Left Ventricular Global Longitudinal Strain in Patients With Surgically Treated Mitral Valve Disease and Preserved Ejection Fraction
Source: Front Cardiovasc Med. 2022 Jan 20;8:775533. doi: 10.3389/fcvm.2021.775533 (PMC8810643; doi:10.3389/fcvm.2021.775533)

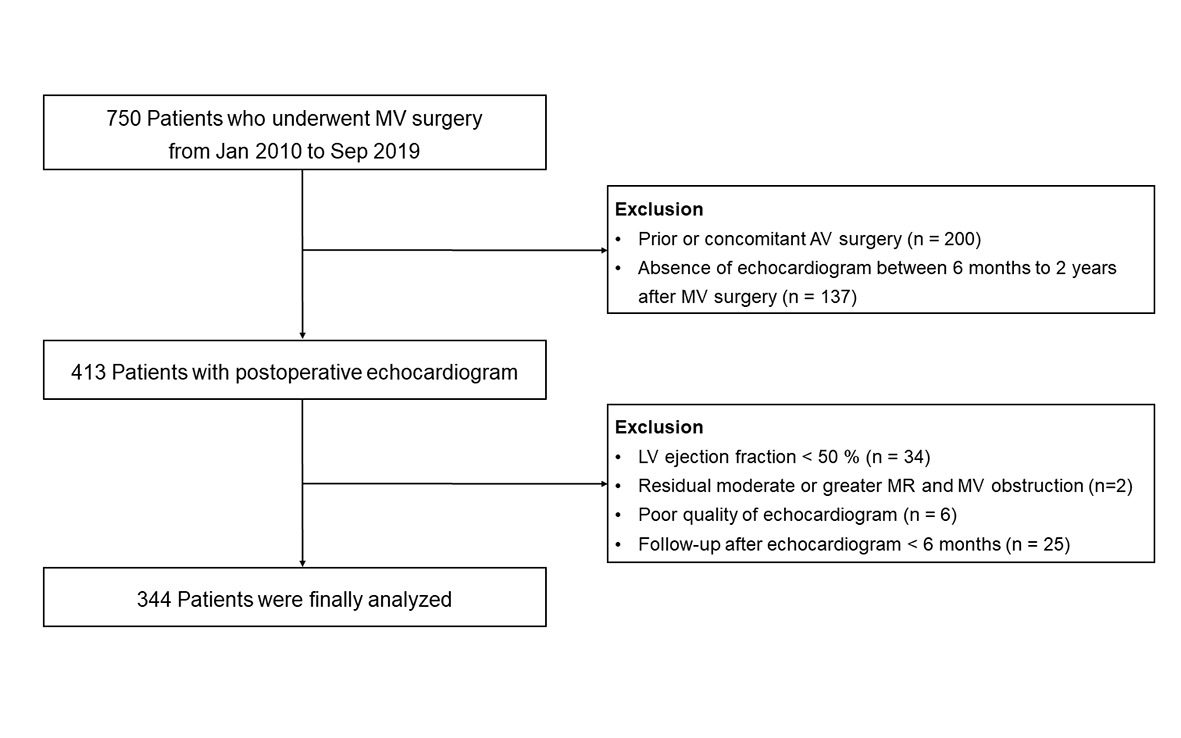

Supplement: Supplementary file 1 [file Image_1.tif]

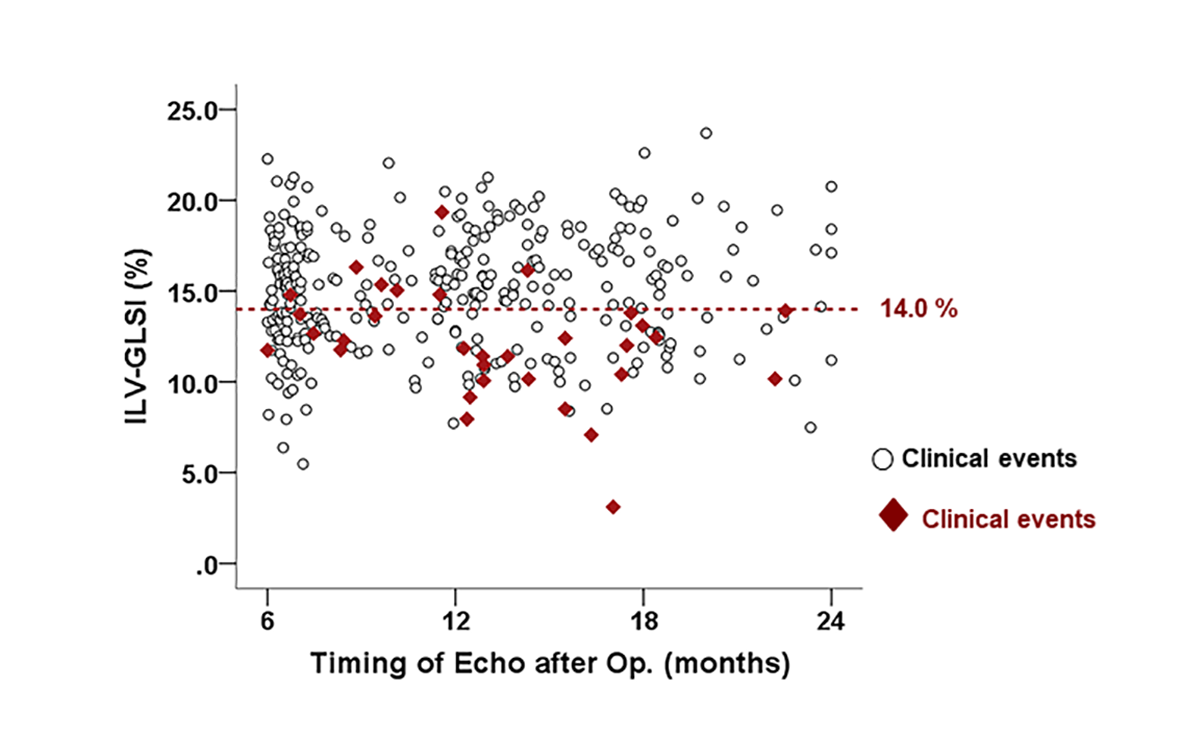

Supplement: Supplementary file 2 [file Image_2.tif]

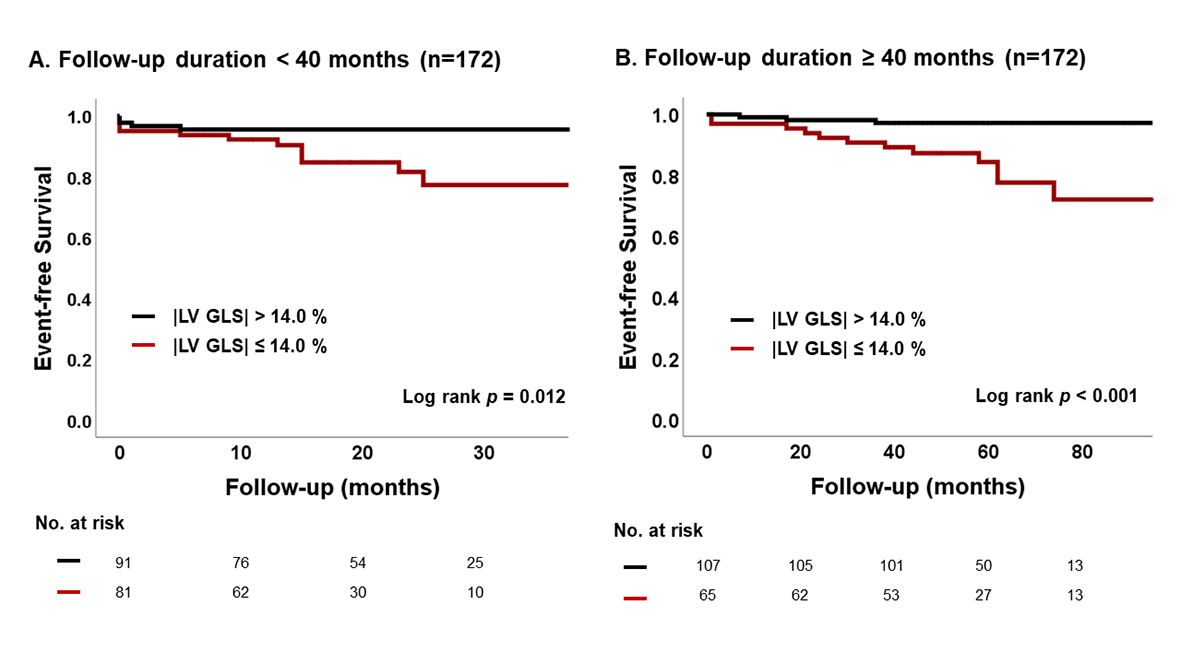

Supplement: Supplementary file 3 [file Image_3.tif]
